# Supplementary material for: The Pea Oligosaccharides Could Stimulate the In Vitro Proliferation of Beneficial Bacteria and Enhance Anti-Inflammatory Effects via the NF-κB Pathway
Source: Foods. 2024 Feb 19;13(4):626. doi: 10.3390/foods13040626 (PMC10887999; doi:10.3390/foods13040626)
Supplement: Supplementary file 1 [file foods-13-00626-s001.zip › foods-2849523-supplementary.pdf]

The components of pea oligosaccharides were analyzed using high performance liquid chromatography (HPLC), and the resulting chromatogram is presented in the Figure S1. The data revealed that sucrose, raffinose, stachyose and verbascose were the primary constituents of pea oligosaccharides with respective contents of 17.15%, 1.15%, 34.53% and 23.82%.

The concentrations of sucrose, raffinose, stachyose and verbascose are determined using the curve equation relating peak area to standard product concentration. The respective standard curves for each substance are as follows:

Sucrose $y = 3591.94*x-326.14$

Raffinose $y = 3214.41*x-136.27$

Stachyose $y = 2630.26*x-18.63$

Verbascose $y=2853.15*x-251.90$

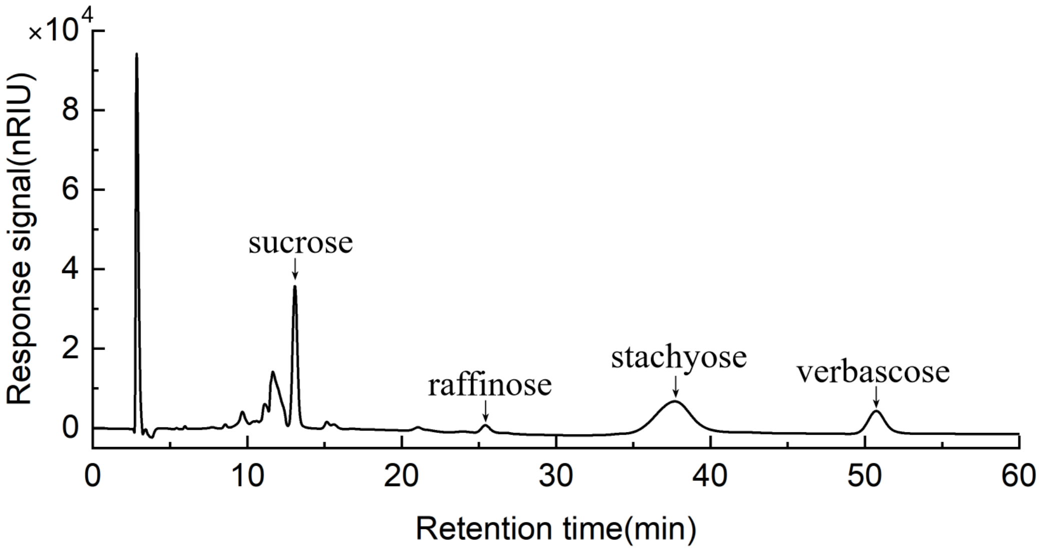

Figure S1. Representative high performance liquid chromatography (HPLC) profiles of pea oligosaccharides.
